# Corneal biomechanics and intraocular pressure assessment after penetrating keratoplasty for non keratoconic patients, long term results

**DOI:** 10.1186/s12886-019-1186-y

**Published:** 2019-08-07

**Authors:** Mohamed Samy Abd Elaziz, Hoda Mohamed Elsobky, Adel Galal Zaky, Eslam Ahmed Maher Hassan, Mahmoud Tawfik KhalafAllah

**Affiliations:** 1 Department of Ophthalmology, Faculty of Medicine, Menoufia University, Shebin El Kom, Egypt; 2 Memorial Institute of Ophthalmology, Giza, Egypt

**Keywords:** Corneal biomechanics, Corneal hysteresis, Corneal resistance factor, Ocular response analyzer, Penetrating keratoplasty

## Abstract

**Background:**

To evaluate corneal biomechanical properties by the Ocular Response Analyzer (ORA) in non keratoconic patients underwent penetrating keratoplasty (PK).

**Methods:**

Corneal hysteresis (CH), corneal resistance factor (CRF), Goldmann- correlated intraocular pressure (IOPg), cornea-compensated IOP (IOPcc) using the ORA, and central graft thickness (CGT) were measured in 30 eyes at least two years after penetrating keratoplasty for non keratoconic indications. IOP using the Goldmann applanation tonometer (GAT) was also obtained after compensation for graft thickness and astigmatism.

**Results:**

The mean age of patients was 33.1 ± 10.13 years; indications for PK were herpetic corneal scar (53.3%), corneal stromal dystrophy (23.3%), traumatic corneal opacity (10%), chemical corneal opacity (6.7%), and Fuchs endothelial dystrophy (6.7%). Mean CH and CRF were 8.52 ± 1.81 mmHg, and 8.56 ± 1.59 mmHg, respectively. Mean CGT was 532.43 ± 30 μm. Mean IOP GAT, IOPg, and IOPcc were 11.88 ± 3.66, 14.64 ± 4.08, and 17.27 ± 4.60 mmHg, respectively (*P* < 0.001). No significant association was found between CGT and IOP readings obtained using either the ORA or GAT. There were significant negative association between CH with both IOP GAT and IOPcc, while CRF had significant positive association with IOPg.

**Conclusion:**

After penetrating keratoplasty for non keratoconic patients, graft biomechanics does not return to average values even 2 years after the operation; moreover, intraocular pressure measurement with ORA gives higher values than thickness compensated GAT.

## Background

Corneal transplantation, regardless the technique, aims at restoration of anatomical as well as optical properties of the eye. Nonetheless, the exact changes in biomechanical properties of the graft are not fully explored. Moreover, intraocular pressure (IOP) follow up after corneal transplantation is of a particular importance, as high IOP (more than 21 mmHg) is reported in transplanted eyes at a high incidence, ranging from 10 to 42% [1, 2].

Using the bidirectional applanation measurements, the ocular response analyzer (ORA; Reichert, Inc., Buffalo, NY) is able to present the four main measurements. Corneal hysteresis (CH) is the difference between the two pressure values, which represents the corneal viscoelastic damping. The mean of these two pressures is the Goldmann-correlated IOP (IOPg). The Corneal-compensated IOP (IOPcc) is a pressure measurement that uses the CH to determine an IO*P* value that is less affected by corneal properties, such as CCT. Corneal Resistance Factor (CRF) is calculated using a proprietary algorithm and is an indicator of the overall cornea resistance [3].

In many reports, CH and CRF were found to be significantly lower in keratoconic eyes than in normal eyes [4, 5]. Anterior segment surgery may change the biomechanical behavior of the cornea as well. Corneal biomechanics (CBMs) showed lower values after laser in situ keratomileusis (LASIK) [6]; also, a transient decline after cataract surgery has been observed [7].

This study aims to evaluate the impact of penetrating keratoplasty on corneal biomechanics for non keratoconic patients.

## Methods

This was a cross sectional study on non keratoconic patients who underwent penetrating keratoplasty at “Ophthalmology department of Menoufia University Hospitals & Memorial institute of ophthalmology in Giza” from January 2014 to February 2016. After receiving the approval of Ethical Committee of the Menoufia University Hospital, all patients received a thorough explanation of the study design and aims followed by a signed informed consent; the study was conducted in compliance with the tenets of the Declaration of Helsinki.

Included eyes showed clear graft after successful penetrating keratoplasty for non keratoconic indications, with all sutures removed; and at least 2 years passed after the operation. The technique for penetrating keratoplasty, carried out by the same surgeon (MSA), was almost identical in all patients with trephine- punch disparity of 0.25 mm (recipient trephine size of 7.5 mm and donor punch size 7.75 mm), postoperative treatment in the early period included topical moxifloxacin hydrochloride 0.5% and prednisolone acetate 1% eyes drops with gradual tapering over the first year; if corneal opacification originated from herpes simplex virus (HSV), additional oral acyclovir was added with a dose of 400 mg twice daily for at least one year.

Sutures removal started, on average, 6 months after the procedure for correction of residual astigmatism, all included eyes had their sutures entirely removed with average of 6 months before measurements.

Exclusion criteria were as follows: keratoconic patients, history of glaucoma, previous intraocular ocular surgery, post keratoplasty corneal scars or opacities, use of contact lenses after PK, and systemic collagen diseases e.g.: Marfan, Ehler Danlos syndromes.

Each subject had a comprehensive ophthalmologic examination, including a review of their medical history, uncorrected distance visual acuity (UDVA), corrected distance visual acuity (CDVA) measured by decimal notation, manifest and cycloplegic refraction, slit lamp biomicroscopy, fundus examination, manifest refraction, and keratometry.

To obtain corneal hysteresis (CH), corneal resistance factor (CRF), Goldmann-related IOP (IOPg) and cornea-compensated IOP (IOPcc); we used the ocular response analyzer (ORA; Reichert, Inc., Buffalo, NY), 3 readings were recorded consecutively at the same session and the average was calculated, all low quality recordings were excluded.

Applanation tonometry measurements (IOP GAT) were done the same day after the ORA readings are obtained, using Goldmann applanation tonometry AT 900 (Haag-Streit, Köniz, Switzerland). Two separate observers took the measurements at 10 min interval, and then the average is recorded. To compensate for corneal thickness; central graft thickness (CGT) was measured separate using an ultrasonic contact pachymeter (PacScan Plus; Sonomed Inc., Lake Success, NY, USA) under topical anesthesia The probe was held perpendicular to the center of the graft and 5 measurements were obtained with the average calculated automatically. Meanwhile the average GAT measurement is manually introduced into the ultrasonic machine to get (thickness corrected IOP) automatically. With corneal astigmatism more than 4 diopters, the prism was rotated so the red mark on the prism holder is set at the least curved meridian of the cornea (along the negative axis).

### Statistical analysis

Data were presented in terms of mean ± standard deviation (± SD), median and range, or frequencies and percentages when appropriate. Paired t-test was used to compare mean IOP readings by the ORA and GAT. Repeated measurement comparison analysis adjusted for multiple comparisons by the Bonferroni method was used to compare mean IOP readings by the ORA and GAT. The agreement between the two tonometers was evaluated with Bland-Altman plots. The correlation of CGT, refractive status (mean keratometric astigmatism and spherical refractive error), and graft biomechanical properties (CH and CRF) with the IOP readings by both tonometers was investigated using multivariate regression analysis. Multiple linear regression analysis models with the ENTER method were conducted with IOPcc and IOPg as the outcomes while age, corneal pathology, CCT, corneal astigmatism, mean K reading, CH and CRF as potential predictors. *P*-value < 0.05 was considered statistically significant. *P* < 0.05 was considered statistically significant.

All statistical calculations were done using computer program SPSS (Statistical Package for the Social Science; IBM Corp., NY, USA) version 21 for Microsoft Windows.

## Results

In this cross-sectional study, a total of 30 eyes underwent PK for non keratoconic reasons, were recruited. Of these eyes, 16 eyes suffered from post herpetic corneal scar, 7 eyes had corneal stromal dystrophy, corneal scars from blunt trauma encountered in 3 eyes, and chemical injuries in 2 eyes. Two eyes suffered end stage Fuchs endothelial dystrophy. The demographic data of this study is summarized in Table 1.

**Table 1:** Distribution of study sample according to Demographic characteristics

Character	No.	(%)
Gender		
Male	14	46.7
Female	16	53.3
Age, (years)		
mean ± SD	33.1 ± 10.13 years	
Median (Min, Max)	30 (20–65) years	

Postoperative spherical equivalent refractive error (SRE), mean keratometry, and keratometric astigmatism were − 4.325 ± 2.16 D, 44.71 ± 2.03 D, and − 6.97 ± 3.21D, respectively. As well, mean CH and CRF were 8.52 ± 1.81 mmHg, and 8.56 ± 1.59 mmHg, respectively. Mean central graft thickness (CGT), measured by ultrasonic pachymetry, was 532.43 ± 30 μm.

As demonstrated in Table 2, there was a significant difference between the 3 IOP measurements: the highest one was IOPcc (17.27 ± 4.61 mmHg) followed by IOPg (14.64 ± 4.12 mmHg), while the least one was thickness compensated IOP GAT as mean (11.80 ± 3.66 mmHg), and that difference was statistically significant (F = 149.04, *p* < .001*).

**Table 2:** Comparison between IOP measurements obtained with the ORA and GAT

IOP measurements (mmHg)	IOP (GAT) (*N* = 30)	IOPcc (*N* = 30)	IOPg (*N* = 30)	Test of significance F	*P*
Mean ± SD	11.80 **±** 3.66	17.27 **±** 4.61	14.64 **±** 4.12	149.04	<.001*
P1	<.001*				
P2		<.001*			
P3			<.001*		

F: One Way Repeated Anovea

P: Significance within 3 IOP measurements; p1 Significance between IOP (GAT) and IOPcc, P2: Significance between IOPcc and IOPg, p3: Significance between IOP (GAT) and IOPg

Sig between measurements assessed by Bonferroni Post hoc test

*: statistically significant

*IOP (GAT)* Intra-ocular pressure (goldmann applanation tonometer)

*IOPcc* Intra-ocular pressure (cornea compensated)

*IOPg* Intra-ocular pressure (goldmann related)

**Fig. 1:**
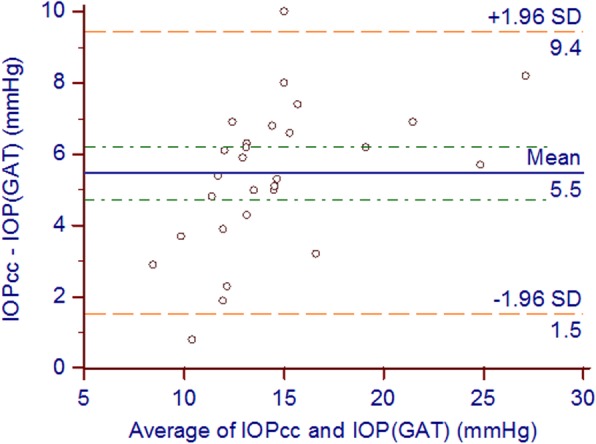
Bland-Altman plots representing the difference between IOP cc and IOP GAT versus the mean of both the red dotted lines represent the upper and lower borders of the 95% limits of agreement

**Fig. 2:**
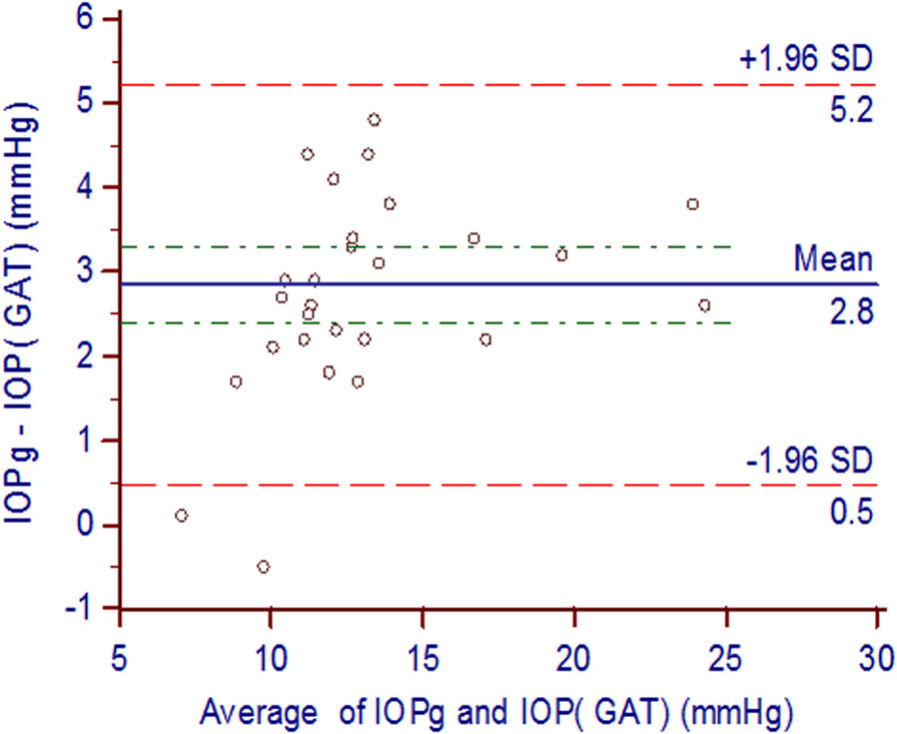
Bland-Altman plots representing the difference between IOP g and IOP GAT versus the mean of both, the red dotted lines represent the upper and lower borders of the 95% limits of agreement

The Bland-Altman plots show the agreement between pressure measurements obtained with ORA and GAT as dots lie within limits of confidence interval (CI) of agreement (Figs. 1 and 2) as the mean difference between IOPcc and IOP GAT was 5.5 ± 2.02 mmHg (95% CI = 4.7 to 6.2 mmHg). While between IOPg and IOP GAT was 2.85 ± 1.21 mmHg (95% CI = 2.39 to 3.29 mmHg).

Correlations of biometric characteristics (central graft thickness, astigmatism, SRE, CH and CRF) with IOP measurements obtained with the ORA and GAT are summarized in Table 3. There was a significant negative association between CH with both IOP GAT and IOPcc, while CRF had significant positive association with IOPg. No significant association was found between CGT and IOP readings obtained using either the ORA or GAT. By using two ways mixed model to assess Agreement between pressure measurements obtained with ORA and GAT (IOP GAT, IOP cc and IOP g) there is statistically significant reliability between 3 measurements as {Intraclass Correlation Coefficient (ICC) average measure {(95% CI) = (.97 (0.94 to 0.98), *P* < .001)}.

**Table 3:** Correlations of biometric characteristics (Central graft Thickness, mean keratometric astigmatism, SRE, CH and CRF) with IOP measurements obtained with the ORA and GAT

Characteristics	Pearson Correlation coefficient (r)	Statistical significance (*p*)
CH vs.		
IOP GAT	−.413	.023*
IOP cc	−.702	<.001*
IOPg	−.357	.052
CRF vs.		
IOP GAT	.36	.051
IOP cc	.05	.794
IOPg	.446	.013*
SRE vs		
IOP GAT	−.094	.62
IOP cc	−.168	.374
IOPg	−.082	.67
Mean Keratometric Astigmatism vs		
IOP GAT	−.157	.41
IOP cc	−.220	.24
IOPg	−.069	.72
Central graft Thickness vs		
IOP GAT	.249	.185
IOP cc	.044	.817
IOPg	.281	.133

*: statistically significant

*CH* Corneal hysteresis

*CRF* Corneal resistance factor

*SRE* Spherical Equivalent Refractive Error

*IOP (GAT)* Intra-ocular pressure (Goldmann applanation tonometer)

*IOPcc* Intra-ocular pressure (cornea compensated)

*IOPg* Intra-ocular pressure (Goldmann related)

Results of the multiple regression analysis models are presented in Table 4. The models were constructed with IOPcc and IOPg as the outcomes while age, corneal pathology, CCT, corneal astigmatism, mean K reading, CH and CRF as potential predictors. Age, CH and CRF were statistically significant predictors for IOPcc and IOPg.

**Table 4:** Multivariate Regression Analysis Models

Variable	IOPcc (Adjusted R^2^ = 0.99)		IOPg (Adjusted R^2^ = 0.99)	
	Standardized Co-efficient (β)	*P* value	Standardized Co-efficient (β)	*P* value
Age	0.04	0.04*	0.05	0.02*
Corneal Pathology	0.002	0.9	−0.004	0.9
Central Corneal Thickness	0.13	0.5	0.02	0.5
Mean astigmatism	−0.003	0.9	0.003	0.9
Mean K	−0.02	0.5	−0.03	0.4
CH	−1.3	< 0.001*	−1.1	< 0.001*
CRF	0.9	< 0.001*	1.2	< 0.001*

*CH* Corneal Hysteresis, *CRF* Corneal Resistance Factor, *IOP cc* Corneal Compensated Intraocular Pressure, *IOPg* Goldman-related IOP, * Statistically significant

## Discussion

The human cornea is a viscoelastic tissue with various biomechanical properties which significantly impact its functional abilities, and ultimately the vision. As well, CBMs can be used as indicators for its structural integrity & IOP measurements. It is now well-established that different physiological factors and corneal disorders alter these properties, which may solve the mystery for many features of these disorders. Age, gender and pregnancy as well as diabetes mellitus, keratoconus, iatrogenic ectasia, Fuch’s dystrophy and keratitis are known to profoundly affect the CBMs [5, 8–13]. Keratoplasty, as a leading therapeutic option for various corneal disorders, induces different changes on CBMs, which may have implications on the functional outcomes [14–16]. Nonetheless, it is underestimated, and scares of literature studied it.

In this study, it is hypothesized that the altered corneal structure in post-PK eyes may result in changes of corneal biomechanics; which in part may be also affected by the recipient remaining corneal tissue. Different corneal layers contribute with different proportions to the biomechanical properties, however, the collagen bundles in the stroma play the master role. Given that, pathological changes affecting the stromal bundles possess the most striking changes in biomechanics [13, 17]. This can explain while different corneal pathologies have their own changes on CBMs, keratoconus is the leading one in which the remaining tissue could pose a major confounder for CBM changes [4, 5, 18–20].

Therefore, we excluded KC patients to get over the primary pathology and highlight the effect of PK on corneal biomechanics as well as IOP assessment. As well, in our series, all sutures were removed at least 6 months before the assessment of individual cases. However, it is worth mentioning that the impact of sutures on CBMs is negligible as reported in different studies [15, 21, 22].

The corneal hysteresis ranges normally between 9.3 ± 1.4 and 11.4 ± 1.5 mmHg, while CRF ranges between 9.2 ± 1.4 and 11.9 ± 1.5 mmHg. In this study, the mean CH and CRF was 8.52 ± 1.81 mmHg and 8.56 ± 1.59 mmHg respectively after two years of successful penetrating keratoplasty. While these values are below the normal range, lack of a self-controlled comparison with the contralateral eyes due to existence of similar corneal pathologies. Comparable to our results, **Murugesan and his colleagues** reported CH and CRF values of 8.4- and 8.8-mmHg respectively with significant correlation detected with IOPg and IOPcc. This report was derived from 100 healthy eyes and 54 post-PK ones with a mean follow-up period of 19 months. Nonetheless, masking the indication (the preoperative pathology) in the PK group brake driving further implications [14].

**Shin et al.** followed 26 eyes for an average of 19 months that underwent PK for various pathologies not including KC. What distinguishes the latter study is the contralateral healthy eye that was assigned as a control. CH and CRF were 8.9- and 10.2 mmHg respectively which are close to our results. However, both of them showed no significant difference between PK and control groups [23].

Glaucoma is among the most common causes of graft failure due to endothelial dysfunction and loss. In addition, the risk of glaucoma after keratoplasty is very high, ranging from 14 to 30%, owing to different mechanisms, including the peripheral anterior synechia which is the most common one for late-onset glaucoma. Given all the previous facts, accurate assessment of IOP after keratoplasty is vital for to maintain functional graft. Goldman applanation tonometer (GAT) is the gold standard tool for IOP Nonetheless, after keratoplasty, surface irregularity and graft thickness as well as the astigmatism pose challenges for this apparently simple measure in practice, with no well-approved tool for this measure [21, 24–26].

In this study, we compared the intraocular pressure measurements obtained from GAT and ORA (IOPg and IOPcc) after PK for non keratoconic patients. GAT showed the least IOP reading while IOPcc gives the highest measurement with a statistically significant difference between the 3 measurements. Outcomes for IOP measurements obtained from different studies after penetrating keratoplasty are summarized in Table 5.

**Table 5:** Comparing published clinical data of IOP assessment after penetrating keratoplasty

Study	Indications for keratoplasty (number of eyes)	Duration after PKP	Results (mm hg)		
			GAT	IOPg	IOPcc
Fabian ID et al. [21]	Different corneal pathologies (51)	65 months (6 to 209 months)	14.2 ± 4.4	15.1 ± 4.2	16.8 ± 4.1
Chou CY et al. [27]	Different corneal pathologies (31)	27.7 months (range 3.0–122.4 months	17.83 ± 5.8	N/A	24.12 ± 8.1
Yenerel et al. [28]	Keratoconus (36)	15 months (range: 15–56 months)	N/A	14.61 ± 2.72	15.46 ± 3.07
Feizi et al. [22]	Keratoconus (45)	At least 6 months	12.2 ± 2.4	15.1 ± 3.5	15.8 ± 3.3
Our study	Non keratoconus (30)	At least 24 months	11.80 ± 3.66	14.64 ± 4.12	17.27 ± 4.61

*GAT* Goldmann applanation tonometer, *IOPcc* corneal-compensated intraocular pressure, *IOPg* Goldmann-correlated intraocular pressure, *SD* standard deviation

*NA* Not available

In addition, we highlight the results of Fabian due to similarities in IOP assessment protocol, including different pathologies and the long mean follow-up period (65 months). Similar to our results, Fabian et al. reported significantly different GAT, ORA and Tonopen IOP measurements with IOPcc was the highest. As well, no correlation was detected for IOP with astigmatism, corneal curvature or graft thickness. However, Fabian reported no correlation between IOP and CBMs except for the inverse correlation between CH and IOPcc (r = − 0.4, *p* < 0.01) and the positive one between CRF and IOPg (r = 0.55, *p* < 0.001), both of them are consistent with our study with a higher negative correlation for IOPcc and CH. This may be explained by the exclusion of KC cases in our study that we believe it profoundly alter the CBMs compared to all other disorders [21].

The concept of no correlation between IOP & CBMs on one side and graft thickness on the other side isn’t odd with many studies reinforce it. **Murugesan et al.** reported no significant correlation between CBMs (CH & CRF) and CCT or astigmatism. As well, IOPcc was the highest reading obtained with an average of 18.6 mmHg similar to our results. However, what is really stunning in Murugesan study is the IOP measurements in normal and post-PK eyes when plotted against graft thickness. According to the Goldman principle, IOP is inversely correlated to the corneal thickness, so in a thicker cornea, IOP is over-estimated and vice versa. Trying to apply this, it is a bit odd to get significantly lower IOP with thicker corneas in the latter study. Normal and post-PK corneas had an average CCT of 530.5 μm and 516.2 μm respectively. The corresponding IOPg and IOPcc were 14.1- and 15.2-mmHg in normal corneas, while in post-PK ones IOPg and IOPcc were 15.9- and 18.6-mmHg respectively [14]. The same concept is highlighted in **Shin’s study** where IOPg and IOPcc was 19.2- and 20.8-mmHg respectively in the PK group compared to 15.07- and 16.2-mmHg respectively in the healthy control eyes. The corresponding CCT was 489.1- and 556-μm in the PK and normal groups respectively [23]. It can’t be more evident that after PK, it is a different situation with various factors behind the scenes.

In penetrating keratoplasty, different elements interact and are supposed to alter the IOP measurement. Astigmatism and corneal curvature are among these factors. Anterior lamellar keratoplasty induces less changes in biomechanics when compared to PK. Building on this, IOP changes are expected to differ from those after PK [26, 29]. Scanning literature, a recent meta-analysis plotted the CBMs changes after PK & DALK including 750 eyes and 218 eyes in both groups respectively. Corneal biomechanics (assessed via CH & CRF) showed no significant changes after DALK in contrast to PK [16]. On the other side, posterior lamellar keratoplasty is well-known for its negligible effect on astigmatism and curvature compared to other techniques, in addition to the overall increase in corneal thickness. **Clemmensen** investigated the IOP & CBMs changes in Fuch’s dystrophy and after DSEAK. IOPcc was significantly higher than GAT in Clemmensen’s study [25]. Similarly, **Vajaranant** explored the IOP after DSEAK with non-contact tonometer, showing that it was independent from the graft thickness [30]. This can delineate how IOP changes are better linked to altered structure and biomechanical changes after keratoplasty rather than changed contour and graft thickness.

Aiming to unveil the key predictors and effectors after PK, the multivariate regression analysis was planned with IOPcc and IOPg as the outcomes while age, corneal pathology, CCT, corneal astigmatism, mean K reading, CH and CRF as potential predictors. The adjusted R [2] for IOPcc and IOPg analysis model was 0.99, which reveal how this model is a well-fitted model in our scenario. Age, CH and CRF were the significant predictors for both IOPcc and IOPg, while CCT had no significant link for either measurement. This analysis model is the first, up to our knowledge, to delineate this relation between IOP on one side and CBMs and CCT on the other side.

While we excluded KC from our study for the previously explained rationale, it is still of value to contrast our outcomes with those obtained from KC studies. **Yenerel et al**. showed that mean CH and CRF values were significantly lower in all groups (manifest keratoconus, forme fruste keratoconus and following PK) when compared to normal eyes. However; they reported mean CH and CRF values after PK of 10.16 ± 1.93- and 9.94 ± 2.34- mmHg respectively [28]. The discrepancy from our results can be partially attributed to the fibrotic effect of wound healing as well as biomechanical characteristics of the transplanted corneal button and to the recipient remaining KC tissue as well. In contrast, our module detected that, even two years after PK, wound healing has a weakening effect on corneal biomechanics in non keratoconic patients. Not far from this, **Feizi et al**. reported a CH and a CRF of 10.1- and 10- mmHg respectively in KC eyes after PK, which is quite different from our results and other studies on non-keratoconic eyes [22]. This explain why KC should be spotted alone in future studies on CBMs.

Limitations of this study is the relatively small sample size considering the underlying multiple subgroups. While this may be explained by excluding KC eyes, the relatively small sample limits the value of the regression analysis model. As well, comparing different corneal pathologies as subgroups couldn’t be conducted. In addition, lack of data for the corneal biomechanics of donors which definitely may have some impact on final postoperative outcomes.

## Conclusion

After PK, the corneal tissue becomes weaker after surgical intervention. Moreover; the cornea doesn’t not achieve the same tensile strength even two years after surgical intervention, with all of its biomechanical measures are compromised. As well, after PK, ORA is valuable for IOP assessment which largely depend on CBMs rather than graft thickness.

## Data Availability

The datasets used and/or analyzed during the current study are available from the corresponding author on reasonable request.

## Abbreviations

CBMs: Corneal biomechanics
CCT: Central cornea thickness
CDVA: Corrected distance visual acuity
CGT: Central graft thickness
CH: Corneal hysteresis
CI: Confidence interval
CRF: Corneal resistance factor
GAT: Goldmann applanation tonometer
ICC: Intraclass Correlation Coefficient
IOP: Intraocular pressure
IOPcc: Cornea-compensated IOP
IOPg: Goldmann- correlated intraocular pressure
LASIK: Laser in situ keratomileusis
ORA: Ocular Response Analyzer
PK: Penetrating keratoplasty
SRE: Spherical equivalent refractive error
UDVA: Uncorrected distance visual acuity
